# Exploring the brain with sleep-related injuries, and fixing it

**DOI:** 10.1093/sleepadvances/zpad007

**Published:** 2023-02-08

**Authors:** Ronald M Harper

**Affiliations:** Department of Neurobiology, David Geffen School of Medicine at UCLA, Los Angeles, CA, USA; Brain Research Institute, David Geffen School of Medicine at UCLA, Los Angeles, CA, USA

**Keywords:** neuromodulation, central apnea, obstructive sleep apnea, blood pressure, sleep, serotonin, single neuron, epilepsy, congenital central hypoventilation, optical imaging

## Abstract

The focus of my research efforts rests with determining dysfunctional neural systems underlying disorders of sleep, and identifying interventions to overcome those disorders. Aberrant central and physiological control during sleep exerts serious consequences, including disruptions in breathing, motor control, blood pressure, mood, and cognition, and plays a major role in sudden infant death syndrome, congenital central hypoventilation, and sudden unexpected death in epilepsy, among other concerns. The disruptions can be traced to brain structural injury, leading to inappropriate outcomes. Identification of failing systems arose from the assessment of single neuron discharge in intact, freely moving and state-changing human and animal preparations within multiple systems, including serotonergic action and motor control sites. Optical imaging of chemosensitive, blood pressure and other breathing regulatory areas, especially during development, were useful to show integration of regional cellular action in modifying neural output. Identification of damaged neural sites in control and afflicted humans through structural and functional magnetic resonance imaging procedures helped to identify the sources of injury, and the nature of interactions between brain sites that compromise physiological systems and lead to failure. Interventions to overcome flawed regulatory processes were developed, and incorporate noninvasive neuromodulatory means to recruit ancient reflexes or provide peripheral sensory stimulation to assist breathing drive to overcome apnea, reduce the frequency of seizures, and support blood pressure in conditions where a failure to perfuse can lead to death.

**Statement of Significance**

The manuscript describes reflections of one investigator on how brain injury in sleep disorders can lead to physiological, cognitive, and affective consequences, and how those outcomes may be overcome using neuromodulatory procedures. The significance rests with realization of the wide-ranging consequences of sleep disorders, and the potential for noninvasive interventions to reduce their impact.

## Introduction

Whatever contributions I made to the sleep field have risen from a combination of extraordinary good fortune and support from a group of talented and generous colleagues and mentors. The focus of my efforts covers a diverse range, from basic neurophysiologic studies and optical imaging of neuronal discharge in brain structures mediating state and vital processes during sleep to magnetic resonance imaging studies of normal and disordered breathing, and more recently, to noninvasive neuromodulatory procedures to overcome these disorders. Every switch in direction has been triggered by appearance of a novel technology or insights from an inspired friend, and none of the goals could have been attained except for prodigious support from many colleagues.

My introduction to the sleep field came during my doctoral training at McMaster University in Hamilton, Ontario, which developed a psychology department heavily weighted to what was called “physiological psychology.” The term “neuroscience” had not yet emerged in the lexicon, and studies of brain actions on behavior were left to those who bridged physiology, neuroanatomy, and behavior in this subset of psychology departments. The McMaster department had drawn former students of Donald Hebb of McGill university, including Cornelius (Case) Vanderwolf and Woodburn (Woody) Heron, who was to chair my doctoral committee. Woody was brilliant, and was exceptionally talented in managing graduate students, essentially leaving them alone to interact with others, and prosper; outside a dissertation defense, I met with Woody about a total of 90 min over 4 years. Case was a model for demonstrating what a behavioral scientist should do- watch their subjects! Case’s contributions to the field are too numerous to mention, but included the description of the relationship of hippocampal theta frequencies to different types of movement, and abolishing long-held concepts of reticular formation arousal processes; his views are well documented elsewhere, for example, Ref. [1].

## Fine Wire Single Neuron Recording (1965–1986)

Among our graduate class was Tom Hoeppner, whose sister was married to John O’Keefe, then a student at McGill, well before he went on to win a Nobel Prize. Tom urged me to visit John to learn his procedure for recording single neurons with bundles of very fine wires in freely behaving animals rather than the etched, stiff microelectrodes that “real” neurophysiologists used. John’s technique was simple, but effective—he cut a bundle of 50 µ wires by pulling them against an embedded hypodermic needle during surgery. I returned to McMaster to face extreme skepticism on feasibility, but we were able to record from freely behaving rabbits during sleep and waking. The discharge patterns during sleep, particularly from thalamic and hippocampal regions, were remarkable, especially when the rabbits entered rapid eye movement (REM) sleep (rare occasions; I soon learned that rabbits, like goats we later studied, have extremely low noise arousal thresholds, and are poor subjects for REM sleep studies!). Observations of the burst-pause patterns in thalamic sites during quiet sleep, and similar patterns with large slow waves under atropine sulfate despite being awake [2] were vivid experiences, comparable to seeing my first REM period in an animal.

**Figure 1.**
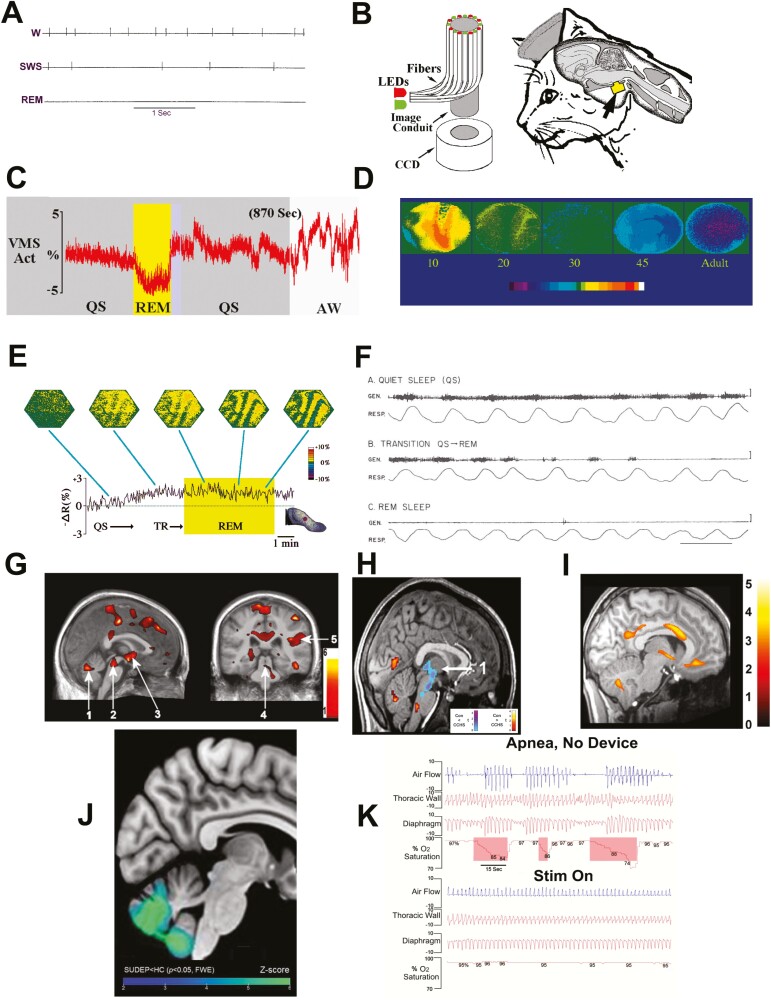
(A) Traces of dorsal raphe neuron discharge across waking (W), slow wave sleep (SWS), and REM sleep; discharge slows during SWS, and nearly ceases during REM (Redrawn from Ref. [3]); (B) Cartoon of coherent fiber optic bundle with surrounding illumination from light emitting diodes (LEDs) and charge-coupled diode (CCD) camera, and placement in the ventral medullary surface (VMS) of a cat (from Ref. [4]); (C) Tracings of mean optical activity of the VMS of a cat during waking (AW), quiet sleep (QS), and REM sleep with depression of activity during REM (Redrawn from Ref. [5]); (D) Averaged VMS activity to pressor challenges in kittens with development in days (from Ref. [6]); (E) Dorsal hippocampal optical traces during quiet sleep (QS), transition to REM sleep showing columnar organization of activity (from Ref. [7]); (F) Tongue genioglossal activity during QS, transition from QS to REM, and during REM sleep (from Ref. [8]); (G) Fractional anisotropy measures in three OSA versus three control subjects showing injury in the cerebellum (left, sagittal-1), midbrain (left-2), and hypothalamus (left-3), midline raphe projecting to the cerebellum (right, coronal-4), and insula cortex (right-5) (from Ref. [9]); (H) Averaged fMRI responses in congenital central hypoventiation (CCHS) subjects, relative to controls, to 5% CO_2_ challenge; reduced responses from the posterior thalamus through the midbrain (1); enhanced activity (warm colors) in the cerebellum and dorsal medulla (from Ref. [10]); (I) Areas of injury in CCHS subjects indicated by increased T2 values. Injuries extending from the rostral through the caudal hypothalamus, the ventral medial frontal cortex, mid- and posterior cingulate, and cerebellum are apparent (from Ref. [11]); (J) Extensive injury (T1 tissue loss, colored regions) to the cerebellum is present in patients with epilepsy who succumb to sudden unexpected death in epilepsy (from Ref. [12]); (K) Periodic breathing (Apnea No Device) is abolished and O_2_ saturations are normalized in a 4-year old CCHS patient with noninvasive stimulation to the tibial nerve (Stim On) from Ref. [13]). A–I, with permission; J–K, open access.

I moved to UCLA and the Sepulveda VA hospital which had an active sleep group—Carmine Clemente, Barry Sterman, and Dennis McGinty; Jerry Siegel joined the group a few years later. We incorporated a variation of a Melzack and Wall procedure to advance bundles of fine wires, allowing fine electrode placement after initial implantation. However, skepticism of the procedure remained. I presented recordings of basal forebrain neurons during sleep onset at a sleep meeting in Bruges; Mircea Steriade, questioning from the floor, was completely disbelieving. Dennis and I later described the suppression of neuronal discharge of dorsal raphe serotonergic neurons during rapid eye movement (REM) sleep [3], which at the time was a unique finding—every other group of neurons studied thus far increased activity during REM. Even the interspike interval discharge of 5-HT neurons differed—a slow, irregular firing pattern during waking and quiet sleep quite unlike the burst-pause pattern of thalamic or other cortical sites (Figure 1A). Being able to assess activity of a defined neurotransmitter system, such as serotonin, with its widespread influences on several respiratory, cardiovascular, pain, and mood systems across sleep and waking states without anesthesia later proved to be invaluable for assessing mechanisms of failure in sudden death in infants and in epilepsy (both state-related phenomena), and in failing behavioral and cardiovascular systems in obstructive sleep apnea (OSA).

## Optical Imaging (1990–2003)

Assessment of discharge patterns of single neurons within structures is useful in evaluating the influence of those sites on behavior. Thus, the high-frequency burst followed by pause pattern observed in thalamic structures during quiet sleep versus the more random, but slow pattern in the dorsal raphe provides insights into the nature of transfer of information between structures. However, neurons within a structure also influence nearby cells, typically in a spatially organized manner. To observe neuronal discharge patterns over a wider area than the field of single neurons, and in a freely moving, drug-free animal, novel procedures had to be developed. Fortunately, an exceptionally talented student, and later, postdoc, David Rector, grasped the significance of directly observing a feature of cell discharge, physical swelling of the neuron, which could reflect imposed light differently, with reflectance depending on displacement of cell size with cell discharge [14]. Visualization of this activity was through a coherent fiber optic bundle surrounded by light-emitting diodes for illumination of cells, with reflected light captured by the fiber optic bundle and a miniature charge-coupled diode (CCD) camera (Figure 1B). Optical imaging of cellular discharge offers multiple advantages, the first of which is to visualize activity at high resolution over a wide area viewed by the fiber optic bundle, which can be in the range of 25 mm^2^^2^ or more. Another strategic advantage is the capability to observe very rapid neural changes; David used the procedure to track the physical displacement that accompanies passage of spike discharges along the course of large axons [15, 16]. The capture rate also extended to very slow changes; John Mayhew and his team visited the lab from the UK, and demonstrated a slow, 0.1 Hz oscillation present in brain parenchyma and microvascular detectable by the probe [17], an aspect of considerable significance when examining slow glial changes associated with migraine and epilepsy.

The optical device could be chronically implanted throughout the brain, and, from a ventral perspective, to the ventral medullary surface (VMS) of feline and goat preparations. We used the latter approach to describe feline VMS responses to sleep state transitions (Figure 1C), hypercapnia, hypoxia, hyperoxia, and hyper- and hypotension [4, 5, 18–24]; the procedure was particularly valuable to follow development of VMS contributions to blood pressure control in early life (Figure 1D). For studies in larger animals, we moved the laboratory essentials to Hubert (Bert) Forster’s laboratory at the University of Milwaukee; Bert raised goats on his home farm for breathing investigations. Goats were of sufficient size to more easily accommodate visualizing the VMS through the then rather large optic probes via a ventral approach requiring placement past the upper airway and esophagus. We followed VMS neural patterns during anesthetic and sleep-waking states during hypoxic, hypercapnic, and blood pressure challenges [25–28], the sleep component being a serious hurdle, since goats, like rabbits, are exceptionally sensitive to arousing stimuli, especially during REM sleep; for that reason, they are often paired with horses to ensure serenity to a prized steed.

The capability to view the spatial organization of discharge within an area can provide insights into the underlying functional organization, including those reflected in electroencephalogram (EEG) activity, such as found in the dorsal hippocampus, by Dave Rector and Gina Poe. Those patterns included marked regional banding as the preparation moved from quiet through REM sleep with its prominent theta activity (Figure 1E) [7, 29, 30]. Morten Kristensen, a graduate student, took advantage of the ability to record from drug-free, freely moving preparations to assess non-pharmacologic stress-related hypothalamic recordings [31, 32]. Ron Frostig, also a graduate student, evaluated information trains of pontine cell discharge [33], and later went on to work with Amiram Grinvald at Rockefeller; together, they used a different principle of optical imaging to visualize activity, with slower capture rates, but highly useful to show cortical columnar organization.

## Infant Sleep Development (1976–1998)

A short time after arriving in Los Angeles, the National Institute of Child Health and Human Development moved to focus research attention on the Sudden Infant Death Syndrome (SIDS) which at that time, was responsible for a staggering number of infant deaths in seemingly healthy infants during the first year of life, occurring silently during sleep for no definable reason. Barry Sterman, Dennis McGinty, and I at the Sepulveda Veterans Administration hospital, and Joan Hodgman and Toke Hoppenbrouwers at Women’s Hospital, University of Southern California, were awarded contracts to evaluate sleep physiology over the first 6 months of life in healthy infants and infants at risk for SIDS, the latter defined as subsequent siblings of SIDS victims. The studies were remarkable; at that time, developmental descriptions of EEG, cardiac and respiratory patterns, and even state duration and arousal characteristics over the first year of life were unavailable, although Arthur Parmelee and his group [34], as well as French investigators [35], had outlined descriptions in the newborn period and in prematurely born infants. The outcomes provided a database that revealed normative development of physiological ultradian rhythms and sleep state development in infants, a unique pattern of heart rate rise in the first few weeks and later decline, EEG descriptions, state periodicity, baseline values for breathing rates and apnea appearances, and subtle, but demonstrable, cardiac, motility, and breathing characteristics in those infants at risk for SIDS [36–54].

Vicki Schechtman, my graduate student and later postdoc, undertook much of the cardiovascular and breathing analyses to demonstrate patterns unique to SIDS, and introduced a number of procedures to examine Poincaré and other distributions of cardiac interbeat and respiratory interbreath intervals useful for distinguishing infants at risk [55–61]. In addition, David Southall in London and Adrian Wilson in Sheffield collected long-term home recordings from over 18 000 infants, some of whom succumbed to SIDS, and provided the recordings for our analyses; these recordings reinforced some of the findings from the UCLA/USC study by pointing to the fixed nature of cardiac and respiratory variability in SIDS victims [55, 57–61]. Tragically, Vicki succumbed to leukemia in the middle of these investigations. Many of the findings of the NICHD collaborative effort were described in a text [62].

The infant studies outlined the importance of normal interactions between physiological processes, especially the loss of dependencies between breathing and cardiac systems, reduction of somatic mobility, and high, and relatively fixed breathing and cardiac rate patterns in considering risk for SIDS. Overall, the findings revealed nervous systems in which a paucity of influences of one system, such as breathing, on another, for example, heart rate or movement. Thus, a rather “brittle” nervous system appeared to be associated with risk for SIDS. Many years later, this pattern of “inflexibility” or loss of connectivity between brain regions would appear in our studies of epilepsy, with affected patients at risk for sudden unexpected death in epilepsy (SUDEP) exhibiting rigidity in influences of one brain area on another when assessed by magnetic resonance imaging (MRI) functional connectivity measures. Structures that should be influenced by other brain areas appeared to have “superhighway-like” traffic to target sites, excluding interrupting extraneous input. As one might guess, that characteristic places an individual at risk, since interrupting an ictal process is made more difficult, and an individual may not adequately respond to ancillary inputs to assist breathing action. However, for both infant breathing failure and epilepsy, that insight of noninfluence from external input opens a potential means for intervention, which we were able to incorporate later in neuromodulatory studies.

## Upper Airway Tone in Sleep and OSA (1976–1985)

Eberhardt (Ebo) Sauerland was at UCLA when I arrived as a postdoc with Carmine Clemente. Carmine had a weekly night-time sleep seminar, fostering numerous interactions among his group, which included Mike Chase, Barry Sterman, Ebo, Dennis McGinty, and me. Mike and I completed a number of studies, including operant conditioning of feline 12–14 Hz (spindle) EEG activity [63]. Ebo encouraged me to study and teach gross anatomy, and we evaluated trigeminal afferent systems [64]. He also had an interest in upper airway muscle activity accompanying swallowing, which he examined by inserting fine wire leads via a hypodermic needle, similar to those I used at McMaster for neuronal recording. Both the trigeminal and upper airway muscle studies were to have remarkable long-term influences on our later research.

In the early 1970s, Christian Guilleminault and his colleagues at Stanford introduced the US sleep field to the dangers of obstructive sleep apnea (OSA), a condition recognized in Europe, especially by Elio Lugaresi, who later went on to describe the neurodegenerative condition of familial fatal insomnia. OSA was an obvious target for us, and Ebo and I studied activity of the genioglossal muscles, the principal protruder muscles of the tongue, and a major airway dilator. We used ourselves, my wife, Becky, and Ebo’s older children in a self-funded study for all-night sleep recordings, and showed the abolition of tone in the tongue muscles during REM sleep (Figure 1F), the vibratory action during heavy snoring, and the potential to exert a major influence on the genesis of OSA [8, 65]. Christian organized a meeting at Ray Kroc’s (of McDonald’s hamburger fame) Santa Barbara retreat on OSA, and included our work. Part of the audience was skeptical. A distinguished and talented sleep and rhythm researcher, Elliot Weitzman, used data from a fiber optic probe during OSA to state repeatedly that “the tongue was not involved;” however, a fiber optic scope mechanically stimulates the heavily sensory-innervated oral airway, reflexively providing tone to the tongue and other airway musculature during apnea, and preventing the collapse that would otherwise occur. Lugaresi, at the end of my talk, ran up and hugged me with a “That’s it!” Elliot later offered me a job at the Einstein School of Medicine which I could not take; unfortunately, our field lost Elliot to leukemia far too prematurely.

Since those early studies, the field has used multiple approaches to dilate the airway through surgical approaches [66], mandibular advancement devices [67], electrical stimulation of the hypoglossal (XII) nerve, the motor nerve to the tongue [68], or pharmacologically, to restore muscle tone [69]. Success of these approaches has varied, with issues ranging from accompanying temporal mandibular joint (TMJ) pain, high cost, and long-term effectiveness. Realization of the critical role in sensory information to maintain sensorimotor circuitry suggested the potential for intervention that we used later.

Although the processes underlying airway collapse in OSA, flaccidity of the upper airway with negative pressure in the thorax by the descending diaphragm, is known, the neural mechanisms underlying that flaccidity during REM have been the object of intense study, especially for neurotransmitter action [70, 71]. It should be noted that OSA also occurs during quiet sleep, deepening the investigation of mechanisms. A failure to activate upper airway musculature before diaphragm descent is a timing issue that involves the cerebellum, a structure for which timing and coordination of motor action is a major function. Cerebellar Purkinje cells, key to timing, are easily damaged by excessive excitation of climbing fibers from the inferior olive, as O’Hearn and Molliver [72] and Welsh [73] demonstrated; for sleep investigators, excessive excitation is a hallmark of hypoxia or ischemia. Those relationships provided a clue that cerebellar injury may contribute to the disrupted timing leading to OSA, but we needed tools that could noninvasively determine those processes. The deep cerebellar fastigial nuclei, recipient of Purkinje neuron input, serve both breathing and blood pressure regulation, and integrity of those nuclei were also a focus; we studied deep nuclei used optical imaging procedures in animal models to evaluate aspects of that control [74].

## Brain Structural and Functional MR Imaging in Sleep-Disordered Breathing; OSA (1985–2022)

Remarkable, and continuing progress has been made by biophysicists in revealing normal and failing brain disruptions with structural and functional MRI (fMRI) procedures. The methodologies allow evaluation of both fibers and gray matter, microstructure of those tissues, functional activity within, and connectivity between areas, and metabolic processes within sites. We began MRI studies with healthy controls, focusing on structures that mediate breathing and blood pressure with inspiratory and expiratory loading, blood pressure, and hypercapnic challenges [75–81], as well as normal development [82, 83].

Initially, the most-viable means to evaluate tissue injury was to assess loss or gain of regional volumes in the OSA brain versus control subjects. We found the expected cerebellar injury in these studies, but also substantial injury in more-rostral areas, including the hippocampus, anterior cingulate, insular cortices, medial frontal cortex, basal ganglia, hypothalamus, anterior thalamus, and mammillary bodies, as well as medullary areas, such as the VMS (Figure 1G) [84–87], all structures that influence the deleterious physiological and behavioral signs found with OSA. The reduced size of the mammillary bodies in OSA [88], as well as the hippocampal injury, was particularly significant in view of the loss of recent memory in the condition, while injury to the insula, anterior cingulate, and cerebellum were expected accompaniments for hypertension, depression [89] and anxiety [90].

Injuries accompanying OSA especially affected both sympathetic and parasympathetic components of the autonomic nervous system, that is, the structures controlling blood pressure, drives for temperature, and multiple hormonal release concerns, for example, glucagon, insulin, and testosterone, among others. The injuries that appeared in the insula, hippocampus, medial frontal cortex, hippocampus, and cerebellum indicated that the hypertension common in OSA likely derived from this damage [91–94]; Some of these injuries were sex-specific [95].

Similarly, functional MR signals that accompanied periodic breathing or central apnea, common in sleep-disordered breathing, was a focus [96], as were responses to expiratory or inspiratory loading [97, 98]. Many of these descriptions are summarized in review form [99, 100].

The recent development of high-speed MR spectroscopic procedures, implemented by our colleague Albert Thomas, and by Rajesh Kumar, initially a postdoctoral fellow, and then a faculty member, allowed assessment of metabolic differences in patients with OSA[101, 102]. In addition, Rajesh and his colleagues demonstrated that the blood brain barrier breaks down in OSA, a finding that likely contributes to neural injury in the condition [103].

## Brain Structural and Functional MR Imaging in Sleep-Disordered Breathing; Congenital Central Hypoventilation Syndrome (2003–2023)

Our first studies in structural and fMRI changes benefited enormously from Jeffrey Alger in the department of Neurology; he was instrumental in studies of OSA and congenital central hypoventilation syndrome (CCHS). CCHS is a rare condition resulting from a genetic mutation (PHOX2B), with the principal signs of loss of sensitivity to CO_2_, major loss to autonomic, especially parasympathetic, regulation, and severe hypoventilation during sleep, and sometimes during waking; individuals typically need mechanical ventilation during sleep [104]. Tom Keens at Childrens Hospital, Los Angeles was a central figure in CCHS research, managed multiple cases from Southern California, and, with his faculty and fellows, Marlyn Woo, David Gozal, and Gabriel Aljadeff, lead the investigative field for CCHS. Marlyn Woo introduced us to David Gozal, a pulmonary fellow who possessed absolutely formidable energy, and pushed both our human and developing feline studies to new heights. David went on to distinguish himself in the sleep, development, and pulmonary fields, establishing an Institute at the University of Kentucky, then served as chief physician and Pediatrics Chair at the University of Chicago, and earned an astonishing number of awards and society appointments, including president of the American Thoracic Society.

Mary Woo from UCLA and Paul Macey, who joined the lab as a postdoc from New Zealand, contributed much to the CCHS studies, which focused on determining what brain areas responded (or not) to CO_2_ and hypoxia, what sympathetic and parasympathetic structures were injured, what leads to the severe anxiety and depression found in these children, and how we could overcome the sleep-related hypoventilation. Remarkably, CCHS children, despite specific physiological deficits of breathing, intolerance to high or low temperatures (breathing difficulties are enhanced with heat, but children often shivered in bed in mid-summer), dysregulation of sympathetic control (excessive sweating to even mild exertion), and emotional behaviors (unawareness of risky challenges, anxiety), were often exceptionally talented and perceptive. Their neural deficits were thus regional and unique. CO_2_ challenges elicited a lack of responses in multiple brain regions, not just the VMS as had been classically proposed, and included cerebellar, posterior thalamic, midbrain, and other areas (Figure 1H) [10]. Low-oxygen and hyperoxia mediation, as well as responses to resistive loading and pressor challenges, were also distributed in multiple areas [105–109]. Structural injury in CCHS children was widespread (Figure 1I) [11, 110], with significant loss of tissue in the insular cortex, hypothalamus, deep cerebellar nuclei, hippocampus, cingulate, and ventral medial frontal cortex, all later described as sympathetic regulatory sites in the human by Kevin Shoemaker [111]. Timing of neural signaling for sympathetic control was also affected, as we found by Jennifer Ogren’s MRI studies to time-tracked maneuvers to pressor challenges [112].

Mary Woo was instrumental in assisting with these investigations, and, because patients with heart failure (HF) share very high rates of sleep-disturbed breathing with conditions we studied, applied similar MRI analyses of physiological challenges to her patients with HF. Those studies found remarkable similarities in injury to those of patients with OSA; in some brain areas, the damage in HF was even more extreme [113–118].

The SIDS studies provoked an interest to find the developmental processes underlying blood pressure control, and for those studies, Luke Henderson, who joined the lab from Richard Bandler’s laboratory at the University of Sydney, traveled with us to the University of Arizona at Tucson that had a high magnetic field animal scanner. We explored fMRI signal changes to serotonin administration, to baroreflex activation, and to hypotension during early and later feline development [119–121], paralleling optical imaging studies of the VMS in the developing kitten to pressor challenges with David Gozal (Figure 1D) [6]. Luke contributed much to the description of fMRI signal changes in OSA, especially to blood pressure manipulation and periodic breathing [92, 96]. He went on to use MRI procedures to outline basic processes underlying migraine pain as well as blood pressure control in his native Sydney.

Of all systems injured in OSA, the sympathetic system is one of the most affected, contributing much to the condition's deleterious consequences, including hypertension, diabetes, reduced osteogenesis, and excessive sweating with accompanying loss of water-soluble nutrients, *e.g*., thiamine and magnesium, necessary to maintain nerve cell integrity. My wife, Becky, and I later described the low levels of these nutrients in OSA [122], and we suspect that the deficient values contribute to the neural injuries found, especially in the mammillary bodies. Patients with HF lose even more water and nutrients (through diuresis, etc.) in addition to the fluid loss accompanying their disordered breathing, aiding the progression to Wernicke-Korsakoff syndrome and wet beriberi often observed in HF.

We used Valsalva, hand grip, and forehead cold pressor challenges to assess sympathetic nervous system action, the last requiring deuterium to avoid water (hydrogen) interference on the fMRI signals. Jeff helped us recruit Rajesh Kumar from India to the lab, who introduced a range of novel MR procedures to show fiber injury and blood-brain barrier damage in sleep-disordered breathing, and disturbances in entropy in epilepsy.

The range of physiologic and behavioral dysfunctions in OSA is extensive; patients are hypertensive, with high sympathetic tone, depressed, anxious, and have severe problems with memory, presumably resulting from intermittent hypoxia, excitotoxic exposure, and breakdown in the blood-brain barrier. Those outcomes, although dangerous in OSA, may be even more severe during sleep-disordered breathing in neonates, with consequences to metabolic control. Eung Pae, who headed the orthodontics program at UCLA before moving to the University of Maryland, was fascinated by the profound injury induced by intermittent hypoxia in newborn life. We showed that simulating the intermittent hypoxia of OSA in immediate post-natal life in rodents damaged the cerebellum [123]; Eung later went on to show exaggerated sympathetic tone, impaired osteogenesis and injury to pancreatic βeta cells with that model. When Covid-19 struck in the second decade, many affected patients unexpectedly developed a form of diabetes. Covid-19, in addition to a range of pulmonary symptoms, often expresses multiple respiratory control problems, including unresponsiveness to high CO_2_ or extreme O_2_ desaturation levels, as we found early in the pandemic [124]. The diabetes appearance may arise secondary to intermittent hypoxia exposure affecting pancreatic β cells in the Covid condition, an example of how compromised breathing can exert far-reaching consequences [125].

The insula exerts significant suppression on sympathetic release by the hypothalamus; if damaged, that control is lost, with resulting hypertension, and transient control of blood pressure affected by damage to the deep cerebellar nuclei. The medial frontal cortex and hippocampal injury will also lead to inadequate regulation of blood pressure since both of the latter structures are part of the circuitry for blood pressure control. The exacerbated sympathetic tone also leads to fragile bones, since high tone interferes with osteogenesis, a particular concern in the elderly, who often develop OSA later in life. The exaggerated sympathetic discharge will also affect glucagon and insulin release, leading to type 2 diabetes. The hippocampal, anterior thalamic, and mammillary body injuries lead to severe short-term memory concerns. The insular, anterior cingulate, and cerebellar injuries, and especially the anterior cingulate damage, likely significantly contribute to depression and anxiety in OSA, affecting half and over a third of patients with OSA, respectively.

## Epilepsy—Basic Animal and Human Studies (1984–2023)

Over the years at UCLA, I maintained a close relationship with the epilepsy group in the Department of Neurology. The initial interactions were mediated through Jerome Engel and Robert Frysinger; Robert arrived as a postdoc in my lab, and we began a series of animal studies that showed the profound effect of the amygdala on breathing, capable of triggering inspiratory efforts on single pulse stimulation, an effect that was abolished on entry into quiet sleep [126]. Gary Sieck and I showed respiratory and cardiac-related discharge of parabrachial pontine neurons; with the periaqueductal gray, a target of amygdala central nucleus neurons [127, 128], and a finding that had implications for later studies in human epilepsy. Ralph Lydic and John Orem showed similar pneumotaxic area respiratory-related discharge [129]. The significance for breathing in epilepsy was not immediately recognized until recent human studies showed that train stimulation of the amygdala triggers apnea, an important consideration for SUDEP because of the common involvement of the amygdala in temporal lobe epilepsy. Gary Sieck later moved to the Mayo Clinic, distinguishing himself with respiratory muscle descriptions, and was President of the American Physiological Society.

During this period, Jing-Xi Zhang, the first neuroscience graduate student to the United States from mainland China, joined the lab. Among a large number of other studies, Jing-Xi found respiratory and cardiac-related neurons in the amygdala central nucleus [130], and blocked the amygdala stimulation breathing influence with a cryoprobe constructed from a Volkswagen fuel injector [131] (I am sad to say, my VW has not worked properly since). He later developed a means to overlay images arising from different modalities for the epilepsy program, an asset essential for the human epilepsy depth electrode program [132]. With Bob Frysinger, we later described neuronal discharge and relationships to breathing and the cardiac cycle in temporal lobe patients who were candidates for surgery for epilepsy [133–135]. Those studies were invaluable later when we studied mechanisms underlying SUDEP.

## Neuromodulation: Sleep, Breathing, and Motor Coupling (2013–2023)

Among the more valuable findings of the sleep-disturbed breathing studies was the recognition that breathing drive did not reflect just CO_2_ sensing, but was driven by numerous other inputs. The inspiration for providing alternative breathing support again derived from Tom Keen’s observations at Children’s Hospital showing that, although CCHS children would slowly turn blue if watching TV until screamed at by their parent to breathe (voluntary breathing is unaffected in CCHS), when let loose on a soccer field, they breathed fine. That coupling of breathing with limb movement is also effective during sleep, even with passive limb movement, as David Gozal’s group showed [136]. Steve Iscoe, now at Queens University in Ontario, who contributed much to our knowledge of control of breathing, laid the groundwork for contributions of other-than-chemoreceptor breathing regulation with a series of studies on somatic input to respiration [137]. John Orem frequently reminded the field of the multiple influences that participate in breathing variability, especially during REM sleep [138]. We showed, using fMRI procedures, that passive foot movement recruited diaphragmatic and foot regulatory regions of the brain (cervical areas for phrenic innervation of the diaphragm, dorsal motor and sensory cortical regions for the foot), as well as cerebellar and pontine coordinating sites, that is, a reflex “coupling” exists between limb movement and breathing [13].

The reflexive recruitment of breathing musculature with leg movement has an ancient origin, derived from at least the time when we were fish and needed to move to oxygenate. Certainly, our species would not have survived if, when we wandered around in a jungle, we encountered a lion, and had to wait around for CO_2_ to build up to enhance breathing for a rapid escape. Tricking the brain into thinking that one is running should be sufficient to enhance respiration. Kalpashri Kesavan and I tested this possibility using premature infants with apnea of prematurity by placing vibratory stimulation on proprioceptors of the feet and hands; the intervention readily lowered apnea, desaturation, and bradycardia rates [139]. The reflex is so old that it reflects a time when we used four limbs for locomotion; vibration of the palm of the hands is sufficient to reduce apnea in paraplegic adolescents (no proprioceptive afferent activity from the lower limbs), as Marlyn Woo and I found [140]. We also applied the procedure to patients with CCHS, and abolished periodic breathing in these patients (Figure 1K).

## SUDEP (2013–2023)

Since a number of ancillary drives to breathing may be lost in patients with epilepsy who are at risk for SUDEP, the sudden, unexpected death of people with epilepsy that occurs principally during sleep, we evaluated brain injury in patients with epilepsy and functional connectivity between sites. Much of our efforts on SUDEP result from collaborations with Beate Diehl and her fellows at University College London. Together with Luke Allen, an exceptionally talented neuroscientist, and a student with Beate, we showed the profound loss of tissue in the cerebellum in those who succumbed to SUDEP, together with lesser declines in those who were at high risk for the fatal event (Figure 1J) [12]. The suspicion is that SUDEP results from a prolonged apnea or extreme hypotension in the postictal period during sleep; we knew from our animal studies and from those of others that the cerebellum, particularly the fastigial nuclei, are critical in providing a “last resort” recovery from either apnea or loss of blood pressure. We also showed damage to other structures essential for maintaining breathing, including the amygdala and periaqueductal gray. The cross-Atlantic interactions continued with a range of studies that lead to descriptions of the microarchitecture of the amygdala and metabolic activity in patients at risk for SUDEP [141–145].

We also found that the subcortical injury in patients at risk for SUDEP was accompanied by volume changes in regional areas within the cortex [146], and that these regions often had a significant role in breathing and cardiovascular influences, a concerning issue for supporting vital functions during and following a seizure. Finally, assessment of tissue injury typically uses a change in local volume as an index; however, both increases and decreases in volume occur, a consequence of damage to neurons, but also injury to surrounding supportive tissue which can swell as well as reduce volume. In-depth evaluation of the latter characteristics typically involves diffusion tensor imaging techniques, procedures which often are not routinely performed clinically for epilepsy, where T1 or T2 scans are the norms. Rajesh Kumar was instrumental in manipulating T1 images from patients with epilepsy to provide indices of entropy, a valuable tool for assessing damage [147]. The universality of T1 image acquisition when evaluating epilepsy provides a unique opportunity to evaluate brain tissue injury from already-acquired images.

The extensive cerebellar injury that occurs in those who succumbed to SUDEP is a concern, because of the “last resort” recovery role of that structure for apnea and blood pressure loss typical of ictal and post-ictal periods. A further cerebellar role exists; direct electrical stimulation of the cerebellum also appears to decrease seizures. Chris De Giorgio from UCLA Neurology and I stimulated proprioceptive fibers that impinge on the cerebellum to reproduce or parallel the electrical stimulation effect. We evaluated a set of mostly patients with Lennox-Gastaut epilepsy to determine if we could use excitation of the remaining healthy cerebellar cells to reduce the frequency of seizures, the principal risk factor for SUDEP, while simultaneously recruiting the reflexive coupling of proprioceptive stimulation to enhance breathing and maintain blood pressure during ictal and postictal periods.

We used a small group of patients, but the outcomes were encouraging. Simple vibration to proprioceptor signals carried by the tibial nerve reduced the frequency of drug-resistant patients at high risk of SUDEP (because of their very high rate of seizures), by up to 46% [148]. This decline, of course, compares favorably with most anti-seizure medications, and these patients were all drug-resistant. Such stimulation, as we found earlier [13], supports breathing and blood pressure, potentially further reducing the risk for SUDEP.

## Migraine Suppression, Sleep Induction, and Breathing Support (2013–2023)

While Becky and I were spending a Christmas holiday with Ebo Sauerland in Reno, Nevada, our discussion turned to means for suppressing migraine pain by vibration. This discussion occurred well before the Nobel committee awarded the 2021 prize for Physiology or Medicine for studies on the role of piezo channels on pain modulation, but our recounting of personal stories related to vibrating chairs and similar stimuli lent credence to a potential means for intervention. Ebo’s wife, Wanda, was suffering from a condition that was accompanied by severe chronic pain. From those discussions evolved a consensus that to modulate pain, we needed to input overwhelming sensory input to a set of cranial nerves using stimuli that were analogous to those proposed by Melzack and Wall for gating spinal pain [149]. Both Ebo and I were familiar with cranial nerve anatomy, and we knew where to direct such input; the auditory canal and surrounding pinna contain sensory fields for cranial nerves 5, 7, 8, 9, and 10, and in the pinna, cervical nerves C2 and C3. From there, we developed silicone impressions of the auditory canals, embedded a vibratory motor, and set out to take control of migraine [150]. On returning to Los Angeles, two neurology colleagues, Frisca Yan Go and Joanna Jen, had subjects for us. The first subject was a second-year premed student who had to drop out of her UCLA studies because of migraine. Her mother, skeptical of these crazies who were attempting to treat her daughter with a buzzing device, flew over from Georgia to Los Angeles to monitor the first trial. Her daughter came in, exceptionally morose, as anyone with a level 8 migraine pain would be. However, after a 30-minute trial, 10 min of which were control periods, she was able to leave with a huge smile, literally skipping out the door. With weekly refresher trials, she resumed studies, and helped guide further device development. Additional trials were also exceptionally successful, including a 14-year-old, also forced to drop out of school, and who had visited over a dozen medical centers across the country which included invasive neurosurgery, all to no avail, and arrived a day after Christmas, 2014, half-carried by a wonderfully patient mother into the lab. It took a half year of refresher trials, with much scheduling by Becky, but she will graduate from MIT in 2023! While visiting from the UK, an engineer described his debilitating migraine attacks lasting 5 days, in which he would lose vision, a condition which persisted for over 20 years. Because of the short visit, we tested the device in the back seat of my car, but he was migraine-free for two years with only one or two 20-minute refresher trials/week before he lost the device (since recovered) to a too-active puppy. A young UCLA neurologist was monitoring a neurosurgical patient with a colleague of mine when an attack began with both pain and vision loss. We intervened immediately; vision was restored in 3 minutes, and the pain was gone in 30 minutes, all while the neurologist tried to continue to monitor the patient.

Several ancillary findings emerged from the migraine studies. The stimulation was perceived by many to be “like meditation.” A number fell into quiet sleep short after stimulation onset, and within 10 min, several of those transitioned into REM sleep, as indicated by peripheral limb twitches, eye movements, and disclosure of dreaming on wakening. Approximately a third fell into at least quiet sleep [151]. Other ancillary benefits emerged, including modulation of breathing and blood pressure [152, 153].

Among the patients with migraine, a few were earlier diagnosed with OSA, and most would also fall asleep during the trial. However, none of those with OSA showed indications of impaired breathing during the trial, despite the obvious signs of quiet sleep. We inferred from that behavior that stimulation of the trigeminal, vagal, and other nerves may have been providing sufficient reflexive tone to the upper airway musculature to prevent airway obstruction, a speculation that was confirmed in isolated pilot cases. The UCLA technology development group provided an Innovation award to study that aspect further, and those studies are continuing.

The outcomes of the proprioceptive stimulation studies on breathing during sleep in premature infants, spinal cord-injured adolescents, and patients with central apnea provide an indication that the nervous systems regulation of breathing during sleep is exquisitely sensitive to other-than-CO_2_ and O_2_ sensory input. Central processes appear to be sensitive to cranial nerve stimulation to control migraine pain, and proprioceptive stimulation to reduce the frequency of seizures. Somatic sensory input may potentially be capable of inducing sleep. It is possible that these neuromodulatory procedures may induce a form of “learning” that can induce long-term neural changes that may be effective with only modest refresher trials to continue the enhanced behavior.

## Summary

Perhaps the most useful contributions from my laboratory have been to identify failing structures in the brain that contribute to dysfunctional processes during sleep and waking, and to suggest interventions to bypass those injured systems. Those interventions typically use readily accessible sensory processes that exert powerful influences on central nervous system activity. Some of the beneficial support processes incorporate old, often untapped reflexes, and many influence actions through cerebellar mechanisms. The potential to assist disorders during sleep through these neuromodulatory procedures is excellent.

## Data Availability

No new data were generated in support of this manuscript, which is essentially a review.

## Notes

This paper is part of the Living Legends in Sleep Research series, which is sponsored by Idorsia Pharmaceuticals and Jazz Pharmaceuticals.

## Brief Biography

Ronald Harper, PhD is a Distinguished Research Professor in the Department of Neurobiology, David Geffen School of Medicine at UCLA, and a member of the Brain Research Institute at UCLA. He served as President of the Sleep Research Society in 1992, and was recipient of the SRS Distinguished Scientist Award in 2010. He served on numerous NIH review committees (NICHD, HL-RIBT, NIDA, ADAMHA, CVRS), and multiple international review groups. His research includes both basic neurophysiological studies in animals and translational studies in humans with sleep-disordered breathing, epilepsy, and migraine. His interests span both early development and adult conditions. The studies have resulted in over 327 peer-reviewed manuscripts, 95 chapters, reviews, editorials, and commentaries, 1 book, 5 patents issued or pending, and 554 abstracts.
